# The History of Medicine and Surgery in Georgia

**Published:** 1894-10

**Authors:** L. B. Grandy

**Affiliations:** Atlanta, Ga.


### THE HISTORY OF MEDICINE AND SURGERY IN GEORGIA.

By L. B. GRANDY, M.D.,

Atlanta, Ga.

Prior to the beginning of this century the practice of medicine and surgery in Georgia, as elsewhere in the United States, was conducted, in the main, by uneducated men. In the original thirteen States of the Union there were about three thousand practicing physicians, and probably not more than two hundred of these had received the doctor's degree from any medical college. These latter, with few exceptions, had graduated in European schools. The territory embraced within our present State limits did not number as many physicians as are now found in the city of Atlanta.

Medical education in those days usually meant an apprenticeship of uncertain duration in some doctor's office, spent in reading the latter's books, which were few, and in compounding his medicines from the raw materials.

There were only four medical schools in the country. The principal one of these was located in Philadelphia. The chair of Practice in the institution was occupied by Dr. Benjamin Rush, whose influence on our early medical history has caused him to be called the "Father of American Medicine." There were not half a dozen medical books by American authors. There was only one medical journal, published quarterly in Philadelphia, so that what little of medical thought there was to record had little opportunity to be recorded.

The practice of obstetrics was yet almost entirely in the hands of women, and the medical man's services were not sought except in abnormal labors when the attending midwives were not equal to the occasion.

The therapeutic tripod of that time was blood-letting, blisters and purgation, applied almost alike in yellow fever, croup and constipation. The greatest of these was blood-letting. There was no god but the lancet, and Dr. Rush was its prophet.

For the early medical history of Georgia, in the eighteenth century, there is no record. There were no books, no journals, no medical societies. But in the last quarter of the century we find two honorable names, distinguished both in our medical and political annals.

When Georgia was still uncertain about joining her sister colonies in their movement for independence, it was Dr. Lyman Hall who was called from the active practice of his profession at Sunbury and sent several times to the Continental Congress in Philadelphia as the representative of St. John's parish. Dr. Noble Wimberley Jones, of Savannah, was also a delegate to this Congress, and an ardent revolutionist. It was through their influence largely that Georgia finally united with the other colonies, making the last of the thirteen States. In 1776 Dr. Hall was one of Georgia's three signers of the declaration of independence, and after the war the first governor of the State (1783). Dr. Hall was one of the few well-educated physicians of his time, and a skillful practitioner of medicine. His political honors were not of his seeking; but he wore them worthily.

After serving two terms in Congress, and the restoration of peace, Dr. Jones resumed the practice of his profession in Savannah (1788). He contributed some papers to medical literature, relating chiefly to the prevalent fevers of his section. In 1795 he was chosen president of the convention by which the original Constitution of the State was amended. He possessed great ability as a physician, and was a man of the highest character, commanding alike the confidence of patients and political constituents.

This passing reference to these patriot-physicians is eminently deserved.

Before 1800 epidemics of smallpox in varying severity had occurred from time to time along our eastern coast, the last in 1779, in and around Savannah. The French and American troops had just evacuated the city and left it and the neighboring country in a very impoverished and unsanitary condition. Smallpox now broke out, and greatly increased the general excitement and distress. At that time inoculation with true smallpox virus was practiced as a preventive measure, but even this could be done only upon an order obtained for that purpose from the royal governor. This was generally refused unless the disease was already present in the household desiring the inoculation. It is much to be regretted that there is no record of the first introduction and practice of true vaccination in the State. It must have been done about 1803 or 1804. In 1802 Dr. David Ramsay, of Charleston, first introduced vaccination into South Carolina, on the person of his own son. We learn * that from him, directly or indirectly, "thousands received the disease." The supposition is that the influence of that vaccination was not long in reaching Georgia, and that the practice was very generally carried out. At any rate, for the next fifty years (1804-1853) there were only five deaths from smallpox in Savannah out of a total from all causes of 14,322.

To our early practitioners the most important and most dreaded disease was yellow fever. It was of frequently recurring interest, and amid much confusion of ideas medical writers, what there were, gave it much attention. Dr. Rush made it the subject of a monograph.

The profession was practically unanimous as to the nature of yellow fever, regarding it not as a disease *sui generis*, but only as the highest in the grade of malarial fevers. But they were greatly divided on its contagiousness and mode of origin.

There were those who believed in its absolute and unqualified contagious character, and these held that the disease was directly or indirectly transmissible from one person to another, like smallpox or measles. Others advocated exactly the opposite view and denied that yellow fever was ever, or under any circumstances, transmissible by a contagious poison from one to another. Nor was there any greater unanimity as to its place and mode of origin.

Among our earliest writers on the subject were Dr. Dennis Smelt of Augusta, and Dr. J. E. White of Savannah. Both were non-contagionists, and both regarded the disease as a malignant form of bilious fever. To these writers yellow fever had no native habitat, but was, in the language of Dr. Smelt,† "the common malady of all hot countries, heightened only by contingent and local causes."

* Ramsay's History of South Carolina. 1809.

† *Medical Repository*, August, September, October, 1805.

The non-contagionists repudiated the idea of importation. In his account of the epidemic in Augusta in 1804, Dr. Smelt denies that it was connected directly or indirectly with any foreign source of infection. Dr. Smelt was not a follower of Dr. Rush. In the matter of treatment he did not like the "monstrous depleting plan." He therefore "bled sparingly, but was liberal in the use of calomel and jalap."

Dr. White was a careful student of yellow fever. He was a close observer, and wrote several good papers on the topography, climate and diseases of Georgia. In one of these papers* he thus expresses his views on yellow fever: "Facts are multiplied to prove the domestic origin of a highly bilious malignant fever in various parts of the United States. It may be called a simple remittent, a bilious remittent, a malignant bilious, or a yellow fever. They are only grades of the same disease, arising from differences of constitution, etc. Accidental or fortuitous causes produce new symptoms, but these do not change the original nature of the disease. At the distance of one hundred miles from the nearest seaport, it is farcical to look for foreign sources of infection. The causes exist in the United States, as in countries within the tropics."

With Dr. White the cause of the disease was always the effluvium arising from decomposing animal and vegetable matter, and he adds, "this septic gas is the product of all countries and places where proper materials and solar heat are found." Whatever we may say of such views now, they were shared then by a large part of the profession, including Dr. Rush. Dr. White believed with Dr. Rush, that all fevers had but one proximate cause—an "irregular action or convulsion in the blood-vessels." He used the lancet freely, and considered it a "divine remedy."

The oldest medical society in the State, and among the first in the country, was the Georgia Medical Society, organized in Savannah in 1804. The objects of the society were "the advancement of medical science, the cultivation of medical ethics, the promotion of good-will among members of the profession, and the restriction of abuses in the practice of medicine." The society met monthly in Savannah. Its membership was confined mainly to that city, but there were

* *Medical Repository*, May, June, July, 1805.

corresponding members in other parts of the State. The society spent much of its time and labor in studying the causes, nature and prophylaxis of the native fevers, from simple febricula to yellow fever. In the swamps and lowlands on both sides of the city indigo and rice were cultivated every year, the latter by the "wet process," or under water. These places were regarded by the local physicians as the sources of their "autumnal fevers." Year after year they urged upon the city authorities the necessity for thorough drainage, but it was not until after the frightful epidemic of 1817 that anything was done. The next spring a "dry culture" ordinance was passed and drainage instituted. "The effect of this measure was immediate, surpassing the most sanguine expectations of its supporters."* The annual mortality, which had been excessive (about one in every fourteen of the population), dropped to one in sixty-two in 1818. But dry culture did not pay. Drainage was neglected, and sanitation grossly disregarded. In 1819 the mortality returned to one in thirteen, and in 1820 it became one in five of the population. Drainage was re-established in 1821, and the mortality for that year was only one in thirty-seven. Whatever may have been the nature of these fevers, whether "yellow," or "autumnal," or "continued," or "malignant malarial," facts demonstrated the position taken by the Georgia Medical Society that the undrained lands about the city afforded a favorable ground for their annual development.

The Georgia Medical Society continued in successful operation for about twenty-five years, when for some reason the interest of members began to wane, and the Society lapsed into inaction.

Bilious remittent fever, so-called, existed side by side with well defined cases of yellow fever. They were often confounded. The former followed the course of western migration, and made its first appearance in the interior of the State, in the country around Milledgeville, about 1807. The Indians had just vacated the section and given place to the white man. The latter cut down the forests and canebrakes, and opened up the soil. Among the settlers was Dr. Tomlinson Fort, who came to practice medicine. He

---

* Antumnal Fevers in Savannah. Dr. W. C. Daniel, 1825.

says:* "Bilious fever appeared as suddenly as the face of nature was changed by the hand of man. For eighteen years it was a formidable epidemic. No tables of mortality were kept, but I cannot be mistaken in placing the deaths from bilious fever alone as high as five per cent. of the whole population each year from 1808 to 1813. This mortality happening in a few months gave to the disease the terror of a pestilence." Dr. Fort was reared in the school of Rush, but while he admired the skill and genius of his great teacher, he did not agree with him on every point. They differed on the treatment of bilious fever. With Rush it was blood-letting and cathartics. Others added blistering and mercurial inunctions. "Such," Dr. Fort says, "was the treatment of bilious fever till about the year 1813." Rush had denounced laudanum and Peruvian bark as either dangerous or useless. Consequently they had been almost abandoned. Dr. Fort was among the first to revive their use and he says, "We found that bark, wine and laudanum were not the poisons they had been thought." He also opposed indiscriminate venesection and salivation. In this section he was not by himself in his efforts to reform and improve existing methods of treatment. He was ably aided by Dr. Charles Williamson and Dr. Samuel Boykin. These gentlemen, all, were country practitioners, successful and prominent in their time and generation, and in the light of their daily experience they searched for a better system of therapeutics. Some of this class were even bold enough to express their distrust of venesection and to question the wisdom of its universal employment. This seems to have been more than Dr. Rush could stand, so he issued his famous "Defense of Blood-letting," which did much to fasten this sanguinary procedure upon the profession for a long time.

The conscientious efforts of reputable physicians to establish a rational basis of therapeutics was contemporaneous with the rise and growth of Thomsonianism, or the Botanic School of Medicine, so-called. This new school made its first appearance in Georgia about 1818, through the agency of traveling representatives of Thomson, who sold his book and the right to use his remedies in the family for twenty dollars. The Thomsonians rejected venesection,

*Dissertation on the Practice of Medicine, p. 65.

blisters, mercurials and metallic salts in general, and relied exclusively on the use of a few vegetable agents, steaming, etc. These vegetable agents consisted mainly of lobelia to "cleanse the stomach," capsicum to "produce internal heat," and a combination of bayberry, hemlock, ginger, pepper and cloves, under the name of "composition tea," which was used to meet a host of miscellaneous indications.

Thomson's followers were as illiterate as himself. The most ignorant people constituted themselves physicians and assumed the practice of medicine on the lines laid down by the New England farmer. Quacks and impostors of the worst sort were quick to take advantage of the cheap opportunities thus afforded to practice physic, and the crimes that were committed in the name of botanic medicine, steaming, etc., though recorded, many of them, were never punished. Thomson himself was tried for the murder of a patient, and some of his followers in Georgia were scarcely more fortunate. Within a few years it became more and more evident that some legislative action would be demanded to protect the people from the consequences of ignorance and fraud. So the legislature of 1825 enacted a law "regulating the licensing of physicians to practice in this State." This was the first medical legislation in the State. The act provided that from and after its passage no person should practice medicine or surgery in the State without proper license; that a person practicing without such license should, upon conviction, be fined a sum not exceeding $500, etc.; that there "shall be established a board of physicians to be assembled annually at the seat of government, who shall, at their annual meeting, examine all applicants; and if, on such examination, they are found competent shall grant to such applicant a license to practice physic and surgery"; that "if any applicant shall have studied and received a diploma from any medical college, said board shall license said applicant to practice without examination." On the first medical board thus provided for there were Drs. Tomlinson Fort, Milton Antony, W. C. Daniel, John Dent, Thomas Hamilton, Charles West, Jas. P. Scriven, Thos. B. Gorman, Alexander Jones, W. N. Richardson, John Gerdine, A. B. Ridley, and others.

 (*To be continued.*)

